# Nuclear Receptor Cofactors in PPAR*γ*-Mediated Adipogenesis and Adipocyte Energy Metabolism

**DOI:** 10.1155/2007/53843

**Published:** 2006-12-19

**Authors:** Emily Powell, Peter Kuhn, Wei Xu

**Affiliations:** McArdle Laboratory for Cancer Research, University of Wisconsin, 1400 University Avenue, Madison, WI 53706, USA

## Abstract

Transcriptional cofactors are integral to the proper function and regulation of nuclear receptors. Members of the peroxisome proliferator-activated receptor (PPAR) family of nuclear receptors are involved in the regulation of lipid and carbohydrate metabolism. They modulate gene transcription in response to a wide variety of ligands, a process that is mediated by transcriptional coactivators and corepressors. The mechanisms by which these cofactors mediate transcriptional regulation of nuclear receptor function are still being elucidated. The rapidly increasing array of cofactors has brought into focus the need for a clear understanding of how these cofactors interact in ligand- and cell-specific manners. This review highlights the differential effects of the assorted cofactors regulating the transcriptional action of PPAR*γ* and summarizes the recent advances in understanding the physiological functions of corepressors and coactivators.

## 1. INTRODUCTION

Peroxisome proliferator-activated receptors (PPARs) are a subfamily of structurally similar members of the nuclear hormone receptor superfamily [1]. However, unlike classical nuclear hormone receptors, PPARs do not bind their ligands with high affinity, but possess a relatively low binding affinity for unsaturated fatty acids and a broad range of compounds that includes eicosanoids and their metabolites (notably prostaglandin PGJ2 and leukotriene LTB4) and synthetic ligands such as fibrates (a drug for treatment of hyperlipidemia) and thiazolidinediones (TZDs, antidiabetic drugs). Thus, these receptors are considered to be nutrient sensors that regulate lipid and glucose metabolism in adipocytes and other metabolically active tissues. PPARs have also been shown to be involved in a diverse array of nonmetabolic functions including inflammation, tissue repair, atherosclerosis, and cancer [2–4].

PPAR*γ* is the most highly characterized member of this subfamily and its regulation by nuclear receptor cofactors will be the focus of this review. Two major splice variants have been found; PPAR*γ*1 is expressed in adipocytes, skeletal muscle, liver and heart tissue, while PPAR*γ*2 is almost exclusively found in adipose tissue [5]. Although PPAR*γ*2 may be more adipogenic than PPAR*γ*1 [6, 7], both isoforms are thought to be essential regulators of adipogenesis [8–10]. A common model for adipogenesis 3T3-L1 cell differentiation into adipocytes is mediated by PPAR*γ*2 [11]. This model has been used extensively to define the relationship between PPAR*γ* and its cofactors. In addition to adipogenesis, PPAR*γ* has been shown to play a role in insulin sensitivity, atherosclerosis, inflammation, and cancer [12, 13].

### 1.1. Overview of cofactors involved in transcriptional regulation of PPAR*γ*

PPAR transactivation is induced by ligand-dependent and independent mechanisms. Ligand-dependent transactivation is induced by ligand binding to the C-terminal activation function (AF-2) domain [14]. The role of transcriptional cofactors in ligand-independent transactivation is poorly understood and outside of the scope of this review. PPARs form heterodimers with the retinoid X receptor (RXR) and bind to PPAR response elements (PPREs) in enhancer sites of regulated genes [15]. In the absence of ligand, nuclear receptor corepressors bind to these heterodimers and recruit histone deactylases (HDACs) to repress transcription. Ligand binding induces a conformational change in the receptor dimer which excludes corepressors from the complex [16]. Ligand binding also increases PPAR's affinity for a number of coactivators, whose binding facilitates chromatin remodeling by histone modification and nucleosome mobilization, leading to the recruitment of the basal transcription machinery to PPAR target genes [17–19]. The short motif LXXLL, where L is leucine and X is any amino acid, is necessary for many coactivators to bind to nuclear receptors [20]. This “NR box” is found in the majority of nuclear receptor coactivators and binds to a hydrophobic pocket in the nuclear receptor binding domain [21].

Cofactors that have been shown to interact directly with PPAR*γ* to initiate its transactivation include members of the p160 family of coactivators, which includes SRC-1/NCoA1, TIF2/GRIP1/NCoA2/SRC-2, and pCIP/ACTR/AIB1/SRC-3 [22]. While having weak histone acetyltransferase (HAT) activities, the C-terminal activation domains of p160 proteins appear to primarily serve as foundations upon which coactivator complexes are assembled. The p160 family of coactivators contains functional activation domains that recruit factors such as cAMP responsive element binding protein (CREB) binding protein (CBP)/p300 via activation domain 1 (AD1). The CBP/p300 complex possesses promiscuous HAT activity, which aids in remodeling chromatin to allow transcriptional activation [23].

The prominent ATP-dependent chromatin remodeling complex SWI/SNF includes components such as BAF250, BAF57, BAF60a, and BRG1 [24]. The SWI/SNF complex is thought to be targeted to nuclear receptor target genes upon ligand induction by interaction with receptors, coactivators, or the general transcription machinery [23]. This complex has also been implicated in chromatin remodeling leading to activation of the PPAR*γ* promoter, thus regulating its expression and adipogenesis [25, 26].

The thyroid receptor associated protein (TRAP)/vitamin D receptor interacting proteins (DRIP)/Mediator complex contains subunits which interact with a variety of transcription factors and serve as a bridge between the basal transcriptional machinery and DNA-bound nuclear receptor cofactors [27, 28]. The TRAP complex interacts with PPAR*γ* in a ligand-dependent fashion. This complex acts more directly on the general transcription machinery, as is evident by its ability to transactivate transcription on naked DNA templates [29]. Furthermore, the TRAP complex interacts with nuclear receptors through PPAR binding protein (PBP)/TRAP220/DRIP205 [30]. Thus, TRAP220 is a critical component of this complex and is required for transcriptional activation of PPAR*γ* [31].

The PPAR-gamma coactivator-1*α*(PGC-1*α*) is a unique PPAR coactivator, which serves as a scaffolding protein to integrate a variety of coactivator [32]. Upon docking to PPAR*γ*, PGC-1*α* recruits HATs such as CBP/p300 and steroid receptor coactivator 1 (SRC-1) to remodel chromatin and initiate transcription [32, 33]. However, interaction of PGC-1*α* and HAT proteins is not sufficient to activate gene transcription; the C-terminal domain of PGC-1*α* also interacts with the TRAP complex through direct association with PBP/TRAP220 to induce transcription (Wallberg et al. [33]). PGC-1*α* has several RNA recognition motifs (RRM), which function in the coupling of transcription to mRNA splicing [34]. The modes of regulation of PPAR*γ* by PGC-1*α* have been reviewed [35, 36].

Although much is known about the mechanisms by which PPAR*γ* recruits coactivators to initiate transcription, considerably less has been demonstrated with regard to transcriptional repression by corepressors. Both NCoR (nuclear receptor corepressor protein) [37] and SMRT (silencing mediator of retinoid and thyroid hormone receptors) [38] directly interact with PPAR*γ* in vitro [39–41]. It may be noted that PPAR*γ* does not appear to be a strong repressor, however, increasing evidence suggests that NCoR and SMRT do repress PPAR*γ*-modulated gene expression during adipogenesis [42, 43].

The exchange of cofactors may be facilitated by nuclear corepressor exchange factors (NCoEx), namely, transducin *β*-like 1 (TBL1) and the related protein TBLR1 [44]. TBL1 and TBLR1 are components of the NCoR corepressor complex [45]. However, they activate PPAR*γ*-dependent transcription in response to rosiglitazone. Moreover, embryonic stem cells with a TBL1 deletion fail to differentiate into adipocytes [46] suggesting that TBL1 is necessary for PPAR*γ* activation. The mechanism of TBL1/TBLR1 activation of PPAR*γ* remains elusive, but is probably linked to the proteasome-dependent degradation of corepressors [46].

### 1.2. Physiological functions of cofactors in adipogenesis

The molecular modes of regulation of nuclear receptor signaling by cofactors have been extensively reviewed [16, 17, 23, 47–49]. Herein we focus on the recent advances in understanding the physiological functions of cofactors in PPAR*γ*-modulated processes, in particular, adipogenesis and energy metabolism. The diversified functions of PPAR*γ* cofactors are studied in cell-based system and/or mice models, which are summarized in Table 1.

## 2. COACTIVATORS

### 2.1. PGC-1*α* a master regulator of adaptive thermogenesis in brown adipose tissue

The thermogenic effect of PPAR*γ* in brown adipose tissue (BAT) is mediated by PGC-1*α*, which is induced by cold and highly expressed in BAT [35, 36]. PGC-1*α* regulates the action of PPAR*γ* on adaptive thermogenesis and fatty acid oxidation by interacting with the PPAR*γ*/RXR*α* heterodimer. This interaction stimulates expression of uncoupling protein 1 (UCP-1), which is responsible for uncoupling *β*-oxidation from ATP synthesis in oxidative phosphorylation, ultimately resulting in the loss of energy as heat [32].

PGC-1*α* is unique in that, in addition to its ligand-dependent binding to the PPAR*γ* ligand-binding domain (LBD), it can also bind to the DNA-binding domain (DBD) and the hinge region of nuclear receptors in a ligand-independent fashion [59]. The ligand-independent binding of PGC-1*α* to PPAR*γ* is mediated by the PGC-1*α* N-terminal domain and results in the expression of enzymes involved in the mitochondrial respiratory chain to activate adaptive thermogenesis [32, 60]. Chromatin immunoprecipitation (ChIP) analyses revealed that the presence of PGC-1*α* decreases the association of corepressors on a PPRE-containing gene in the absence of exogenous ligand without altering the binding of PPAR*γ*, and PGC-1*α* is sufficient to recruit SRC-1, p300, and RNA polymerase II to the PPRE-containing gene in the absence of rosiglitazone [61].

The ectopic expression of PGC-1*α* in white adipose tissue (WAT) in vitro causes induction of the genes associated with the brown fat phenotype, such as UCP-1 and components of the electron transport chain [62, 63]. The presence of UCP-1 in WAT is associated with a more brown-fat like phenotype, enhanced metabolic rate and insulin sensitivity, and resistance to obesity [64–66], which could indicate a potential therapeutic role for PGC-1*α* and UCP-1.

**Figure 1:**
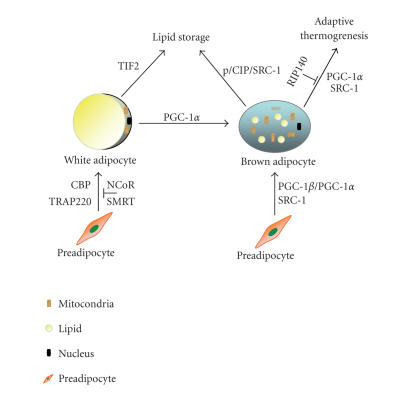
Putative functions of PPAR*γ* cofactors in white adipose- and brown adipose-modulated lipid and energy metabolism. Positive regulators are highlighted in red. Preadipocytes can be differentiated into white adipocytes via transcriptional regulation of PPAR*γ* by coactivators CBP and TRAP220, or differentiated into brown adipocytes via transactivation by PGC-1*β*, PGC-1*α*, and SRC-1. TIF2 plays roles in lipid storage from white adipocytes, while p/CIP and SRC-1 function to promote lipid storage in brown fat. PGC-1*α* is not only involved in adaptive thermogenesis but it also promotes the conversion of white adipocytes into brown adipocytes. SRC-1 is the only member of p160 proteins that show clear function in energy expenditure.

The function of PGC-1*α* in adaptive energy metabolism is reinforced in the PGC-1*α* knockout mouse model [53]. PGC-1*α* null mice are born with no obvious defects during embryonic development but have reduced mitochondrial function. Intriguingly, null mice are lean and resistant to diet-induced obesity. The lean phenotype is largely due to hyperactivity caused by lesions in the striatal region of the brain which controls movement [53]. The closely related family member PGC-1*β* has been less studied, but it appears to induce mitochondrial biogenesis and fatty acid oxidation in several cell types [67–69]. Thus, PGC-1*β* can regulate some but not all activities of PGC-1*α*. The most recent PGC-1*β* knockdown studies in immortal preadipocyte lines derived from PGC-1*α* null mice reveal complementary actions of the two PGC-1 proteins [52]. Loss of PGC-1*α* alone severely impairs the induction of thermogenic genes but does not affect brown fat differentiation (Figure 1). Loss of either PGC-1*α* or PGC-1*β* exhibits a small decrease in the differentiation-induced mitochondrial biogenesis; however, double knockdown results in a reduced number of mitochondria and functional defects [52]. This study implicates that PGC-1*β* plays a role in brown fat differentiation, and is at least as important as PGC-1*α* in this process (Figure 1).

### 2.2. Effects of the p160 coregulators SRC-1, TIF2/SRC-2, and p/CIP/SRC-3 on energy metabolism and homeostasis

Members of the 160 kd protein family of coactivators are able to interact directly with the AF2 domain of PPAR*γ* to allow nuclear receptor transactivation function in a ligand-dependent manner via an *α*-helical LXXLL motif on p160 protein's N-terminal domain. Furthermore, CBP/p300 interacts with p160 cofactors and directly with PPAR*γ*, possibly providing additional stability to the complex through an increased number of contact points [70]. However, although CBP/p300 binding is required for maximal PPAR*γ* activity in vitro, minimal data exists showing a requirement for these cofactors in adipogenesis [71].

Mice deficient in p160 family members exhibit very different phenotypes, providing insights into their physiological functions in adipogenesis and energy metabolism [50]. TIF2^−/−^ mice exhibit enhanced adaptive thermogenesis and protection against obesity, whereas SRC-1^−/−^ mice are predisposed to obesity with accompanying reduced energy expenditure [50]. TIF2^−/−^ mice also show improved metabolic profiles and increased whole-body insulin sensitivity [50]. TIF2 seems to have a greater influence on the p300/PPAR*γ* complex than does the SRC-1 complex, which could possibly be attributed to a weaker capacity of SRC-1 to interact with other coregulators such as p300/CBP and TRAP220, as these coregulators have been shown to have roles in adipogenesis [31, 71]. An increase in lipolysis is observed in TIF2^−/−^ cells, indicating a reduced potential for the storage of fatty acids. Furthermore, a TIF2 dose-dependent attenuation of the PGC-1*α*/PPAR*γ* activation complex in the presence of SRC-1 suggests that TIF2 competes with SRC-1 for the formation of PGC-1*α*/PPAR*γ* complexes. However, TIF2 does not significantly enhance PPAR*γ* transactivation mediated by PGC-1*α*, and an increase in PGC-1*α* expression level was observed in BAT of TIF2^−/−^ mice [50]. Thus, TIF2 appears to be linked to WAT differentiation and fat storage by potentiating PPAR*γ* activity (Figure 1). In contrast, SRC-1^−/−^ mice displayed increased fat mass and plasma leptin levels. Moreover, the mRNA of UCP-1, PGC1*α*, and AOX were decreased in BAT, suggesting that the thermogenic machinery in BAT is diminished in the absence of SRC-1. Thus, SRC-1 largely contributes to brown fat differentiation and energy expenditure in brown fat (Figure 1).

A recent study involving p/CIP^−/−^ SRC-1^−/−^ double knockout (DKO) mice revealed that p/CIP and SRC-1 are required for induction of genes necessary for adaptive thermogenesis and lipid storage in BAT [51]. These DKO mice consume more food, both on chow and high fat diets, as a result of decreased blood leptin levels; however, the DKO mice are resistant to diet-induced obesity and remain lean when compared to single knockout and wild type littermates. Furthermore, these mice are more physically active and have increased basal metabolic rates. This phenotype appears to be the result of failed induction of PPAR*γ* target genes, resulting in increased basal metabolism and decreased adipogenesis [51]. Although p/CIP single knockout mice do not exhibit a strong phenotype in adipogenesis, p/CIP appears to potentiate SRC-1-mediated fat storage in BAT and perhaps adaptive thermogenesis (Figure 1).

### 2.3. The SWI/SNF chromatin remodeling complex is required for induction of the PPAR*γ* promoter and adipogenesis

The mammalian SWI/SNF (mating type switching/sucrose nonfermenting) family of ATP-dependent chromatin remodeling enzymes plays critical roles in the activation of PPAR*γ* transcription for adipogenesis. The core components of the complex include either the Brg1 or Brm ATPase and several Brg1/Brm-associated factors (BAFs). Although in vitro analyses of SWI/SNF complexes containing Brg1 or Brm reveal similarities in chromatin remodeling [72], differences in their functions have been observed in vivo. Brg1 knockout mice are embryonically lethal, and heterozygotes show a predisposition for tumor development [73]. In contrast, Brm knockout mice and cells show only a slight difference in proliferation from wild type [74].

PBAF, a multisubunit complex containing Brg1 and BAF180 subunit was shown to activate PPAR*γ* transcription in an in vitro chromatin-based system [75]. The necessity of the SWI/SNF chromatin remodeling complex is illustrated by experiments revealing that Pol II and general transcription factors are dissociated from the PPAR*γ* promoter when cells are transfected with dominant negative components of the SWI/SNF complex [25]. This suggests that function of the SWI/SNF complex is essential to formation of the preinitiation complex (PIC) on the PPAR*γ*2 promoter and subsequent transcription initiation. Expression of dominant negative Brg1 or hBrm leads to blocked induction of the PPAR*γ* activator and adipogenesis, which was measured both morphologically and by expression of two adipogenic marker genes, aP2 and adipsin [25]. Because Brg1 and hBrm are both crucial members of the SWI/SNF chromatin remodeling complex, this evidence suggests that the SWI/SNF enzymes are required for the activation of PPAR*γ* and adipogenesis [25].

BAF60c, another component of the SWI/SNF complex, serves to anchor the SWI/SNF complex to PPAR*γ*. GST pull-down experiments as well as co-IP confirmed the ability of BAF60c to interact with PPAR*γ*. Moreover, BAF60c interacts with PPAR*γ* in a ligand-dependent fashion to enhance the transcriptional activity of the receptor [26]. However, BAF60c was not shown to affect adipocyte differentiation in these experiments suggesting that BAF60c is not the only factor docking SWI/SNF to PPAR*γ* [26].

### 2.4. TRAP220/DRIP205/PBP is required for transactivation of PPAR*γ*2 and adipogenesis

The TRAP complex has been implicated as a general transactivator of nuclear receptors [76], apparently functioning by direct interaction with DNA-bound activators and RNA polymerase II [30]. Appreciable evidence for the TRAP complex serving as a coactivator for PPAR*γ* is derived from an in vitro transcription assay in which purified TRAP complex significantly enhanced the transcriptional activity of PPAR*γ*2 on a PPRE-template. GST pull-down assays confirmed the ability of the TRAP complex to bind PPAR*γ*2 only in the presence of TRAP220 [31]. Thus, TRAP220, also known as DRIP205 and PBP [77], anchors the TRAP complex to PPAR*γ* target promoters. A TRAP220^−/−^ mutation is embryonically lethal at day 11.5, showing defects in vascular development similar to those in PPAR*γ* ^−/−^ mice, indicating that TRAP220 function is nonredundant and essential for development [54, 78]. Studies using immortalized TRAP220^−/−^ MEFs reveal that TRAP220 acts as a coactivator for PPAR*γ*2 and is an essential mediator of adipogenesis [31]. TRAP220^−/−^ cells exhibit defective PPAR*γ*2-stimulated adipogenesis and expression of adipogenic marker genes. These adipogenic defects can be rescued by ectopic expression of TRAP220 [31]. These data support the model that TRAP220 acts as an anchor in TRAP complex binding, and may also play a role in binding to the CBP-associated complex.

### 2.5. Evidence of a megacomplex in PPAR transactivation

PPAR interacting protein PRIP/NRC/RAP250/TRBP is ubiquitously expressed in adult mice, and binds to PPAR*γ* enhancing ligand-dependent transcription [55, 56, 79]. PRIP is also necessary for embryonic vascular development, as well as normal cardiac and neural development, as shown by a lethal null mutation [56, 57]. Mouse embryonic fibroblasts isolated from these PRIP null mice exhibited a decreased capacity for ligand-dependent transcriptional activation of PPAR*γ* [56, 57]. PRIP interacting protein with methyltransferase domain (PIMT) was isolated in a yeast two-hybrid screen using PRIP as bait and enhances PRIP-mediated PPAR*γ* transactivation [80]. Interestingly, PIMT binds to CBP/p300 and TRAP220 supporting a model in which the TRAP complex anchored by TRAP220 is bound to PPAR at the same time as the CBP/p300-associated complex [81].

The isolation of PPAR*α*-interacting cofactor (PRIC) complex which enhances the transcription of PPAR*α* further supports the existence of megacomplex on PPAR-target gene promoters [82]. Of the 25 polypeptides comprising PRIC complex, 18 contained one or more LXXLL motifs. Recognized proteins identified in the PRIC complex include SRC-1, CBP, TRAP220, PRIP, PIMT, TRAP100, and PGC-1, suggesting that CBP-associated complex and TRAP220 bound basal transcription factors may be bound simultaneously. PRIC285, a novel member of the PRIC complex renamed PPAR DNA-binding domain interacting protein (PDIP-1), was shown to bind to the DBD of PPAR*γ* in a yeast two-hybrid assay. Two splice variants, PDIP-1a and PDIP-1b, were identified, and both were shown to transactivate all three isotypes of PPAR and thyroid receptor, whereas PDIP-1a but not PDIP-1b transactivates estrogen receptor (ER) *α* and androgen receptor (AR), indicating some receptor specificity [82].

## 3. COREPRESSORS

### 3.1. Corepressor RIP140 regulates energy metabolism but not adipogenesis

RIP140 was originally identified as a corepressor of ligand-dependent ER function by binding to the AF-2 domain [83]. It was later shown to bind to PPAR*α* in a yeast two-hybrid screen [84]. Although PPAR*γ* and RXR ligands promote the interaction of RIP140 with rat PPAR*γ* in solution, RIP140 interaction with PPAR*γ*/RXR heterodimers does not occur on DNA. This cofactor downregulates the activity of several nuclear receptors specifically by attenuating transactivation mediated by SRC-1. For instance, RIP140 competes with the coactivator SRC-1 for binding to PPAR*γ* [84]. This evidence is suggestive of a model in which RIP140 indirectly regulates the activity of PPAR*γ* by competing with coactivators such as SRC-1. RIP140^−/−^ mice exhibit upregulation of energy metabolic genes UCP-1 and carnitine O-palmitoyl transferase I (CPT-I) and increased *β*-oxidation in adipocytes, albeit adipogenesis is unaffected [58]. This data suggests that a highly specific set of PPAR*γ* mediated functions is modulated by RIP140 repression while other PPAR*γ* functions such as adipogenesis remain unaltered.

### 3.2. Transcriptional corepressors for PPAR*γ*: NCoR and SMRT

NCoR and SMRT function to recruit HDAC (histone deacetylase) complexes, which covalently modify nucleosomes to compact DNA and repress transcription [47]. Binding of NCoR and SMRT to NRs is mediated by the corepressor nuclear receptor box (CoRNR) [85]. This motif is very similar to the NR box with a consensus sequence of hydrophobic residues including leucine and isoleucine [86, 87]. The *α*-helix that contains the CoRNR box is predicted to be longer than the helix containing the NR box in coactivators [87], presenting a possible mechanism for cofactor selection via the ligand-induced conformational change of the NR. Thus, conformational change may exclude corepressors from the AF-2 binding pocket.

Evidence exists suggesting that in the absence of ligand, PPAR*γ* recruits the transcriptional corepressors NCoR and SMRT to downregulate PPAR*γ*-mediated transcriptional activity. Gene silencing of NCoR or SMRT in 3T3-L1 preadipocytes has been shown to increase adipocyte differentiation, a classical PPAR*γ*2 function [42]. Moreover, treatment with the synthetic PPAR*γ* ligand pioglitazone decreases both PPAR*γ*-SMRT and PPAR*γ*-NCoR interactions, although the PPAR*γ*-SMRT interaction decrease is much more prominent. Furthermore, in a separate study by Krogsdam et al., repression of PPAR*γ*-mediated transcription by NCoR exists even in the presence of ligand [88]. These studies underscore the transcriptional repression of PPAR*γ* by NCoR and SMRT in vivo.

It appears that gene-specific factors may affect the conformation of PPAR*γ*, further complicating the ligand-receptor-repressor interaction. One example of this variability is the differential activation of glycerol kinase (GyK) and aP2 transcription. Although both contain PPREs, PPAR*γ* recruits corepressor NCoR to the GyK gene while recruiting coactivators to the aP2 gene [89]. The addition of TZD results in the activation of GyK by recruiting PGC-1*α* and displacing NCoR, while TZD treatment has little effect on transcription of aP2 and does not recruit PGC-1*α* to the aP2 promoter [89]. These data suggest that gene-specific PPAR*γ* receptor conformation leads to the recruitment of different cofactor complexes.

Another corepressor, Sirt1, has also been shown to effectively inhibit PPAR*γ*-mediated transcription [90]. This NAD-dependent deacetylase binds to NCoR and SMRT, presenting a model where Sirt1 is recruited to PPAR*γ* via interactions with NCoR and/or SMRT. This was further supported by loss of Sirt1-mediated repression when NCoR levels were decreased via RNAi [90].

### 3.3. Summary of coactivators and corepressors in lipid and energy metabolism

Cellular energy metabolism is maintained through a delicate balance between energy intake and energy expenditure. When energy intake exceeds energy expenditure, excess energy is stored as lipid in WAT. Although BAT also allows storage of small amount of lipids, it is mainly responsible for energy dissipation. As PPAR*γ* plays an essential role in lipid homeostasis, it is not surprising that multiple PPAR cofactors are involved in lipid and energy metabolism; namely, processes including adipocyte differentiation, lipid storage, and adaptive thermogenesis (Figure 1). PPAR*γ*/RXR heterodimers are master regulators of preadipocyte differentiation into brown and white adipocytes. Multiple lines of evidence support the model that CBP/p300 and TRAP220 participate in white adipocyte differentiation, and this process is reversibly regulated by corepressors NCoR and SMRT [31, 42, 71]. On the contrary, differentiation of preadipocytes into BAT is regulated by a different set of coactivators such as PGC-1*β*/PGC-1*α* and SRC-1 [50, 52]. Conversion of white adipocyte to brown adipocyte-like cells can be at least partially catalyzed by ectopically expressed PGC-1*α* [62]. TIF2 plays important functions in the storage of fatty acids in WAT as evident by the fact that TIF2^−/−^ mice are protected from obesity and TIF2^−/−^ cells show an increase in lipolysis [50]. Brown adipocytes are enriched in mitochondria and the major function is adaptive thermogenesis in rodents. PGC-1*α* and SRC-1 are positive regulators of the thermogenic capacity of BAT [50, 52, 53], whereas the corepressor RIP140 appears to negatively regulate this process [58]. Lipid storage in brown adipocytes can be regulated by coactivators p/CIP and SRC-1 [51]. Figure 1 summarizes some of the major players in lipid and energy homeostasis based on current literature. It is worthy to note that some cellular processes require more stringent regulation than others, such that more than one member of the closely related proteins are simultaneously involved. For example, complementary actions of p/CIP and SRC-1 in lipid storage of brown adipocytes and two PGC-1 coactivators in brown fat differentiation are absolutely essential.

### 3.4. Ligand- and promoter-specific coregulator recruitment in PPAR*γ* transactivation

A comparison of natural and synthetic PPAR*γ* ligands reveals a distinct differential recruitment of transcriptional coactivators. 15d-PGJ2, an endogenous PPAR*γ* ligand, is capable of inducing interactions between the PPAR*γ*/RXR heterodimer and SRC-1, TIF2, p/CIP, p300, and TRAP220 [91]. However, the synthetic PPAR*γ* ligand troglitazone did not induce interaction between the PPAR*γ*/RXR heterodimer and any of these coactivators. Furthermore, the transactivation function of PPAR*γ* was shown to be increased by these coactivators in the presence of 15d-PGJ2 and 9-HODE, but not troglitazone. FK614, a non-TZD synthetic PPAR*γ* ligand, and two TZDs, rosiglitazone and pioglitazone, induce recruitment of SRC-1, CBP, and PGC-1*α* when bound to PPAR*γ*. However, the level to which SRC-1 and CBP are recruited by FK614-bound PPAR*γ* is altered in comparison to rosiglitazone- and pioglitazone-bound receptor (Fujimura, 2005) while PGC-1*α* showed similar levels of recruitment. These data suggest specific ligands can differentially define the coactivator complex, and that similar coactivators might have distinct in vivo functions.

## 4. CONCLUSIONS

The race to find new nuclear receptor coactivators and corepressors has resulted in a rapid increase in the number of known cofactors accompanied by insufficient knowledge as to their mechanisms of interaction and transcriptional mediation. Initial investigation has shown that seemingly redundant or promiscuous cofactors have a high amount of context specificity. Gene sequence- and ligand-specific nuclear receptor conformation appears to affect cofactor complex recruitment. The relative expression levels of coactivators and corepressors modulate nuclear receptor transactivation. In the case of PPAR*γ*, there are only a few examples of these differential conditions thus far. Further investigation of these interactions may eventually allow for a better comprehension of context-specific expression profiles. Partial PPAR*γ* agonists, such as FK614, that differentially activate PPAR*γ* target genes may be effective in treating metabolic disease while reducing the side effects (e.g., promoting obesity) caused by current TZD-based treatments. The ability to target unique expression profiles may also lead to a more widespread ability to treat illnesses related to nuclear receptor function.

## Figures and Tables

**Table 1:** Loss-of-function studies on PPAR*γ* cofactors in adipogenesis and energy metabolism

PPAR*γ* cofactor	Phenotype in the absence of the cofactor	

	Cell-based studies	Mouse studies

*Brg1, hBrm (SWI/SNF components)*	Blocked adipogenesis (Salma et al. [25])	—
	Reduced presence of Pol II and GTFs on the promoter (Salma et al. [25])	
	Decreased PPAR*γ* transcription (Salma et al. [25])	

*TIF2*	Increased lipolysis (Picard et al. [50])	Enhanced adaptive thermogenesis (Picard et al. [50])
		Protection against obesity (Picard et al. [50])
		Increased insulin-sensitivity (Picard et al. [50])
		Improved metabolic profile. Increased lipolysis (Picard et al. [50])
		Decreased presence of PPAR*γ*

*SRC-1*	—	Predisposition to obesity (Picard et al. [50])
		Reduced energy expenditure (Picard et al. [50])
		Reduced fatty acid oxidation in brown adipose tissue (Picard et al. [50])
		Decreased energy expenditure, attenuated fatty acid oxidation (Picard et al. [50])

*SRC-1/pCIP double knockout*	Abrogated preadipocyte differentiation (Wang et al. [51])	Diminished lipid storage in brown fat; increased caloric intake on both chow and high-fat diet due to increased leptin levels; resistance to diet-induced obesity; increased basal metabolic rate and energy expenditure (Wang et al. [51])
	Reduced expression of PPAR*γ*-target genes, including UCP-1, due to corepressor recruitment and decreased PPAR*γ* recognition of PPREs (Wang et al. [51])	

*PGC-1*α**	Impaired induction of thermogenic genes in BAT (Uldry et al. [52])	Reduced mitochFondrial function (Lin et al. [53])
	Decreased number and impaired function of mitochondria (Uldry et al. [52])	Resistance to obesity and hyperactivity (Lin et al. [53])

*TRAP220/DRIP205/PBP*	Defective PPAR*γ*-stimulated adipogenesis (Ge et al. [31])	Defective vascular development similar to that seen in PPAR*γ*-null mice (Barak et al. [54]; Zhu et al. [55])

*PRIP/NRC/RAP250/TRBP*	Decreased PPAR*γ*-mediated transcriptional activation (Antonson et al. [56]; Zhu et al. [57])	—

*RIP140*	Upregulation of genes involved in energy dissipation (Poweka et al., 2006)	Increased oxygen consumption and resistance to high-fat diet-induced obesity (Leonardsson et al. [58])
	Increased PGC-1*α* expression (Poweka et al., 2006)	Expression of lipgenic enzymes is decreased. UCP-1 (involved in energy dissipation in BAT) expression is increased (Leonardsson et al. [58])

*NCoR and SMRT*	Increased adipocyte differentiation (Yu et al. [42])	—

*Sirt1*	Decreased NCoR levels (Picard et al. [43])	—

## LIST OF ABBREVIATIONS

15dPGJ2: 15-deoxy-Δ 12, 14-prostaglandin J2
9-HODE: OX-LDL, 9-hydroxy-10, 12-octadecadienoic acid
ACTR: Activator of thyroid and retinoic acid receptor
AF: Activation function
AIB1: Amplified in breast cancer 1
AR: Androgen receptor
BAF: Brg1/Brm-associated factor
BAT: Brown adipose tissue
CBP: CREB-binding protein
ChIP: Chromatin immunoprecipitation
CoRNR: Corepressor nuclear receptor box
CPT-I: Carnitine O-palmitoyl transferase I
CREB: cAMP-responsive element binding protein
DBD: DNA-binding domain
DKO: Double knockout
DRIP: Vitamin D-interacting protein
EMSA: Electrophoretic mobility shift assay
ER: Estrogen receptor
GRIP: Glucocorticoid receptor interacting protein
GST: Glutathione *s*-transferase
GyK: Glycerol kinase
HAT: Histone acetyltransferase
HDAC: Histone deacetylase
HMT: Histone methyltransferase
LBD: Ligand binding domain
LTB4: Leukotriene B4
MEF: Mouse embryonic fibroblast
NAD: Nicotinamide adenine dinucleotide
NCoA: Nuclear coactivator
NCoEx: Nuclear corepressor exchange factors
NCoR: Nuclear corepressor
NR: Nuclear receptor
NRC: Nuclear hormone receptor coregulator
p/CIP: p300/CBP interacting protein
PBP: PPAR binding protein
PDIP: PPAR DNA-binding domain interacting protein
PGC: PPAR-gamma coactivator
PGJ2: Prostaglandin J2
PIC: Preinitiation complex
PIMT: PRIP interacting protein with methyltransferase domain
PPAR: Peroxisome proliferator-associated receptor
PPRE: PPAR-response element
PRIC: PPARα-interacting cofactor
PRIP: PPAR interacting protein
PRMT: Protein arginine methyltransferase
RAP: Receptor-associated protein
RIP140: Receptor interacting protein 140
RRM: RNA-recognition motif
RXR: Retinoid X receptor
Sirt1: Sirtuin 1
SMRT: Silencing mediator of retinoid and thyroid receptors
SRC: Steroid receptor coactivator
SWI/SNF: Mating type switching/sucrose nonfermenting
TBL1: Transducin *β*-like 1
TBLR1: Transducin *β*-like related 1
TIF: Transcriptional intermediary factor
TRAP: Thyroid receptor-associated protein
TRBP: Thyroid receptor-binding protein
TZD: Thiazolidinedione
UCP-1: Uncoupling protein 1
WAT: White adipose tissue
Wy-14643: (4-Chloro-6-[(2, 3-dimethylphenyl)amino]-2-pyrimidinyl) thioacetic acid
